# Medication errors related to high-alert medications in a paediatric university hospital – a cross-sectional study analysing error reporting system data

**DOI:** 10.1186/s12887-023-04333-2

**Published:** 2023-10-31

**Authors:** Sini Kuitunen, Mari Saksa, Justiina Tuomisto, Anna-Riia Holmström

**Affiliations:** 1 HUS Pharmacy, HUS Helsinki University Hospital, Helsinki, Finland; 2 Division of Pharmacology and Pharmacotherapy, Faculty of Pharmacy, University of Helsinki, Helsinki, Finland; 3 Tuulos Community Pharmacy, Tuulos, Finland

**Keywords:** High-alert medication, Hospital, Medication error, Medication error reporting, Medication management and use process, Medication safety, Patient safety, Paediatrics, Risk management

## Abstract

**Background:**

Paediatric patients are prone to medication errors, and only a few studies have explored errors in high-alert medications in children. The present study aimed to investigate the prevalence and nature of medication errors involving high-alert medications and whether high-alert medications are more likely associated with severe patient harm and higher error risk classification compared to other drugs.

**Methods:**

This study was a cross-sectional report of self-reported medication errors in a paediatric university hospital in 2018–2020. Medication error reports involving high-alert medications were investigated by descriptive quantitative analysis to identify the prevalence of different drugs, Anatomical Therapeutic Chemical groups, administration routes, and the most severe medication errors. Crosstabulation and Pearson Chi-Square (χ2) tests were used to compare the likelihood of more severe consequences to the patient and higher error risk classification between medication errors involving high-alert medications and other drugs.

**Results:**

Among the reported errors (*n* = 2,132), approximately one-third (34.8%, *n* = 743) involved high-alert medications (*n* = 872). The most common Anatomical Therapeutic Chemical subgroups were blood substitutes and perfusion solutions (B05; *n* = 345/872, 40%), antineoplastic agents (L01; *n* = 139/872, 16%), and analgesics (N02; *n* = 98/872, 11%). The majority of high-alert medications were administered intravenously (*n* = 636/872, 73%). Moreover, IV preparations were administered via off-label routes (*n* = 52/872, 6%), such as oral, inhalation and intranasal routes. Any degree of harm (minor, moderate or severe) to the patient and the highest risk classifications (IV-V) were more likely to be associated with medication errors involving high-alert medications (*n* = 743) when compared to reports involving other drugs (*n* = 1,389).

**Conclusions:**

Preventive risk management should be targeted on high-alert medications in paediatric hospital settings. In these actions, the use of intravenous drugs, such as parenteral nutrition, concentrated electrolytes, analgesics and antineoplastic agents, and off-label use of medications should be prioritised. Further research on the root causes of medication errors involving high-alert medications and the effectiveness of safeguards is warranted.

**Supplementary Information:**

The online version contains supplementary material available at 10.1186/s12887-023-04333-2.

## Background

Medication errors (MEs) and other adverse drug events are significant factors jeopardising patient safety in hospitals [1, 2]. The most important development areas include risk management focusing on medication-related factors (e.g., the use of high-alert medications), provider-and patient-related factors (e.g., high-risk patient groups, such as very young children), and systems-related factors (e.g., high-risk care environments, such as university hospitals providing the most complex medical treatment) [2–4]. Especially paediatric patients are prone to MEs and other adverse drug events [2, 3, 5–7]. The potential for adverse drug events within paediatric inpatient populations is about three times as high as among adults, and it is estimated that one in four children experiences an adverse drug reaction during hospitalisation [5, 8]. In a recent study investigating serious patient safety events in 44 children’s hospitals, more than 20% of severe accidents were caused by MEs [6].

High-alert medications are drugs with a heightened risk of causing significant patient harm when used in error [9]. Some international medication safety organizations have identified high-alert medications and related medication safety risks by using self-reported ME reports of healthcare organizations, harmful errors described in the literature, studies that identify the drugs most often involved in harmful errors, and other input from healthcare practitioners and safety experts (e.g., [9–11]). Moreover, self-reported MEs have been used as research material in studies investigating high-alert medications in different hospital settings (e.g., [12–14]). The purpose of identifying high-alert medications and related medication safety risks is to strengthen the medication management and use (MMU) process by implementing safeguards to prevent MEs related to these drugs, make errors visible, and mitigate harm [3, 9, 15, 16].

Some research focuses on investigating high-alert medications from the perspective of paediatric patients [12, 14, 17–21]. Most of these studies have aimed to create a list of paediatric high-alert medications in hospitals [17, 18, 20, 21] or intensive care unit settings [19] using a survey [17, 19, 21], literature search [17], Delphi technique [18], or an analysis of ME reports [20]. Two studies [17, 21] have also aimed to identify safety measures for ME prevention and one [20] to identify contributing factors of MEs. High-alert medications have also been associated with the most serious MEs in paediatric hospitals [14] and neonatal intensive care unit (NICU) [12] settings. Still, fewer studies have focused on describing the characteristics of paediatric MEs involving high-alert medications [12, 14]. Consequently, this study aimed to investigate the prevalence and nature of self-reported MEs on high-alert medications in a paediatric university hospital. The study also explored whether MEs related to high-alert medications were more likely associated with severe patient harm and higher error risk classification when compared to other medications.

## Methods

### Study design

**Fig. 1:**
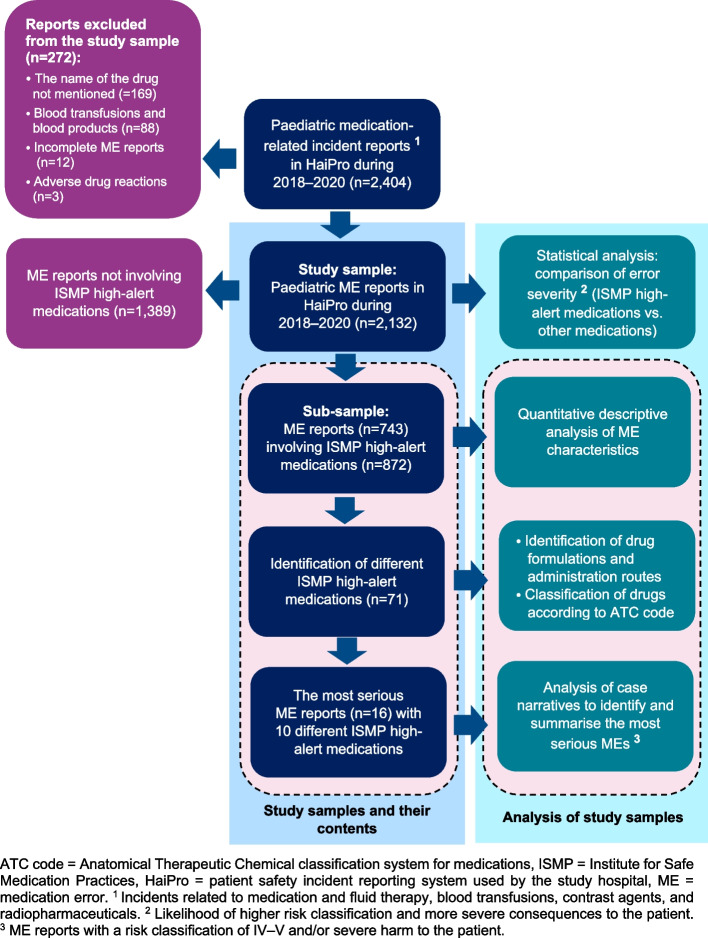
Outline of the study

This cross-sectional study employed a descriptive quantitative analysis of ME reports related to high-alert medications in paediatric hospital settings (Fig. 1, Table 1). When possible, the STROBE (Strengthening the Reporting of Observational Studies in Epidemiology) checklist for cross-sectional studies was applied in the reporting of the study (Supplementary File 1) [22]. The study material was retrospectively collected from register-based voluntary ME reports from a paediatric university hospital. The key definitions of this study are described in Table 1 [9, 23–25].

**Table 1:** Definitions of key concepts

Key concept	Definition
Medication error (ME)	‘Any preventable event that may cause or lead to inappropriate medication use or patient harm while the medication is in the control of the healthcare professional, patient or consumer’ [23]. Both ME reports that occurred to the patient and near misses intercepted before reaching the patient were included. The study sample did not include adverse drug reactions from an appropriate medication management and use (MMU) process [24]
Anatomical Therapeutic Chemical (ATC) Classification System	In the ATC classification system, drugs are divided into 14 main groups and four sub-levels depending on the target organ or group of organs and their chemical, pharmacological and therapeutic properties [25]
High-alert medications	High-alert medications are drugs with a heightened risk of causing significant patient harm when used in error [9]. Although mistakes may or may not be more common with these drugs, the consequences of an error are usually more devastating to patients

### Study setting

Our study occurred in the Department of Children and Adolescents at the HUS Helsinki University Hospital (HUS), Finland. The department is responsible for paediatrics, paediatric surgery, paediatric neurology, and child psychiatry activities in the capital area of Finland. Also, the most difficult patient cases (e.g., heart surgeries, organ transplants, severe cancer cases) and rare diseases from all over Finland are treated in the current setting. The department includes wards for different paediatric specialities, neonatal and paediatric intensive care units, an anaesthesia and surgery unit, two paediatric emergency departments, outpatient infusion clinics, home hospital services, and several outpatient clinics. Patients are mainly under 16 years of age. At the time of the study, the department had 234 beds in two hospital sites.

### Data collection and processing

The study material consisted of a census sample of medication-related patient safety incident reports recorded in the Department of Children and Adolescents at HUS during 2018–2020 (*n* = 2,404) (Fig. 1). The respective time frame was selected for the data extraction as it represented a steady time between moving to new facilities (2018) and implementing a new electronic health record system (late 2020) in the hospital. The data was extracted from HaiPro, a voluntary and anonymous electronic reporting system for the patient- and medication-safety incidents in Finland [26, 27]. In the study site, an average of 804 paediatric ME reports per year have been reported during 2015–2022. All hospital staff members can submit ME reports comprising structured information (drop-down-variables for case notifier’s working unit, time, place, nature, and type of the incident) and open-narrative information (free-text description of what happened and how the event occurred, consequences and circumstances of the event, contributing factors, and case notifier’s view on error prevention) on errors. After submission, responsible persons (usually a senior doctor and an assistant head nurse) in each care unit code the reports according to an established structured classification system. These persons are trained for coding and supported by the paediatric quality manager and Quality and Patient Safety unit of HUS.

The classification system for ME reports in HaiPro includes variables such as the medication involved in the error, event nature (e.g., an actual error or a near miss), professional group of the case notifier, the consequences to the patient (no harm, minor harm, moderate harm, severe harm, not known), incident type (e.g., prescribing error, preparation error, administration error), and risk classification [26, 27]. The risk classification of ME reports is determined on a scale of I to V (I = insignificant risk, II = low risk, III = moderate risk, IV = significant risk, and V = serious risk). It is based on the combination of 1) the consequences of the injury to the patient (I = very minor, II = minor, III = moderate, IV = significant, V = severe) and 2) the likelihood of error recurrence (I = rare, II = unlikely, III = possible, IV = probable, V = almost certain). The risk classification is used for identifying events posing a high risk (IV–V) of harm and recurrence for further analysis in the healthcare organisation using HaiPro. MEs, including near misses, reported during the study period were extracted from the HaiPro database to a Microsoft Excel spreadsheet.

The medication-related incident reports (*n* = 2,404) were manually searched (SK, MS, JT) to include all ME reports suitable for inclusion (Fig. 1, Table 1). High-alert medications were identified according to the Institute for Safe Medication Practices (ISMP) list of high-alert medications in acute care settings (Table 1) [9], because it is widely used internationally in hospitals and has also been applied in paediatric settings [12, 14, 26]. However, it is noteworthy that some of the ISMP acute care high-alert drugs were not used in our pediatric department regularly (e.g., direct oral anticoagulants and factor Xa inhibitors, direct thrombin inhibitors, insulin U-500, opium tincture, oral sulfonylurea hypoglycemics, oxytocin) during data collection, and were consequently not regarded in the present study [9]. In reports where the medication was not reported structurally, this information was supplemented by identifying the medication(s) from the case narrative. In addition, the drug formulation and route of administration of potential high-alert drugs were identified, as these properties may affect the high-alert medication status (e.g., ME reports related to amiodarone were included only if the drug was administered intravenously) [9]. In cases where it was difficult to determine whether a high-alert medication was involved, other supporting literature was used [25, 28]. A consensus discussion was held between the researchers to decide on inclusion (SK, MS, JT, A-RH). All study material was imported to SPSS Statistics 25.0 (SPSS Inc., Chicago, IL, USA) to perform statistical analysis.

All different high-alert medications (*n* = 71) identified within the sub-sample of ME reports (*n* = 743) were classified according to the Anatomical Therapeutic Chemical (ATC) classification system (Fig. 1, Table 1) [25]. In addition to the readily available structured data in the ME reports (e.g., event nature, risk classification), the researchers (SK, MS, JT) manually searched other key variables (administration route and drug formulation) from the case narratives. Finally, the most serious cases were recognised by searching ME reports, which involved the highest risk classifications (IV–V) or had caused severe harm to the patient.

### Descriptive quantitative analysis

A descriptive quantitative analysis reporting frequencies (n) and percentages (%) was performed on the readily available structured data and manually searched variables within the sub-sample of ME reports (*n* = 743) related to high-alert medications (*n* = 872) (Fig. 1). First, the basic characteristics of the included ME reports were extracted to describe the study sample. After that, the prevalence of different high-alert medications was determined to identify the most abundant medications and ATC groups. We also identified the drug formulations and administration routes most often associated with the ME reports related to high-alert medications. Finally, characteristics of the most serious ME reports (risk classification IV–V and/or MEs causing severe harm to the patient) were summarised.

### Statistical analysis

A statistical analysis was performed to compare error severity between the sub-sample of ME reports including high-alert medications (*n* = 743) and ME reports related to other drugs (*n* = 1,389) within the study sample (*n* = 2,132) (Fig. 1). Cross-tabulation was used to compare the likelihood of higher error risk classification and more severe consequences to the patient between ME reports involving high-alert medications and other drugs. For both variables, the missing data were addressed by grouping ME reports with missing values under the “not reported” classification. In HaiPro, there is also a separate classification (“not known”) for situations where neither the case notifier nor personnel responsible for coding the ME reports is aware of the consequences to the patient. Both of these classes of “not reported” and “not known” were included in the analysis. The statistical significance was tested by using Pearson Chi-Square (χ2) test. *A p-value* of 0.05 was selected as the level of statistical significance.

### Research ethics

This study was a retrospective register-based document analysis from ME data collected to organizational quality improvement purposes in HUS. A study approval was obtained from HUS. According to Finnish National Board on Research Integrity, ethical approval is not needed for retrospective register-based study unless there is a special risk for information security in merging data or it is a medical study [29]. This study was not a medical study that intervened to patient’s physical or mental integrity according to definition of Finnish Act on Medical Study (1999/488). The study employed anonymous error reporting system data, so the results cannot be linked to specific individuals, such as patients or employees. The research material was handled and stored confidentially so that only the members of the research group who signed the confidentiality and data protection agreement had access to it.

## Results

### Characteristics of the ME reports comprising high-alert medications

Among the study sample of ME reports (*n* = 2,132), 34.8% (*n* = 743) of the ME reports were related to high-alert medications. The majority of MEs involving high-alert medications reached the patient (*n* = 469/743, 63.1%) and were reported by registered nurses (*n* = 423/743, 56.9%) (Supplementary File 2). The MEs involving high-alert medications had been observed most often in paediatric wards (*n* = 423/743, 56.9% vs. other drugs *n* = 850/1,389; 61.2%). However, a greater proportion of MEs associated with high-alert medications were reported in neonatal intensive care unit (*n* = 142/743, 19.1% vs. other drugs *n* = 210/1,389; 15.1%) and in paediatric intensive care and monitoring unit (*n* = 68/743, 9.2% vs. *n* = 51/1,389, 3.7%). The reports usually involved errors in administration (*n* = 300/743, 38.3%) or prescribing (*n* = 160/743, 20.4%). A more detailed description of the characteristics of the ME reports in the sub-sample comprising high-alert medications (*n* = 743) compared to ME reports related to other drugs (*n* = 1,389) is reported in Supplementary File 2.

### High-alert medications involved in ME reports

Among the ME reports included in the sub-sample (*n* = 743), 71 different high-alert medications (total *n* = 872) were identified (Fig. 1, Table 2). These were classified into 14 level 2 therapeutic subgroups and further into 26 level 3 therapeutic subgroups according to ATC codes (Table 3). Almost 40% of the identified high-alert medications belonged to blood substitutes and perfusion solutions (B05), of which intravenous solutions (B05B) and intravenous solution additives (B05X) were the most represented subgroups. The second most common ATC level 2 subgroup was antineoplastic agents (L01, 15.9%), followed by analgesics (N02, 11.2%), antithrombotic agents (B01, 8.6%), and cardiac therapy (C01, 7.9%).

**Table 2:** High-alert medications (*n* = 872) and administration routes were identified in the sub-sample (*n* = 743 medication error reports)

High-alert medication	Administration route	n (%)
Parenteral nutrition preparations	IV	130 (14.9)
Hypertonic sodium chloride (greater than 0.9%)	IH ^a^, IV, PO ^a^	93 (10.7)
Potassium chloride concentrate	IV, PO ^a^	66 (7.6)
Morphine	IV, PO	47 (5.4)
Heparin	IA, IV	43 (4.9)
Oxycodone	IM, IV, PO	42 (4.8)
Vincristine	IV	33 (3.8)
Fentanyl	IV, SL	28 (3.2)
Methotrexate	IV, IT, PO	27 (3.1)
Enoxaparin	SC	26 (3.0)
Cytarabine	IV, IT	23 (2.6)
Lipid emulsion	IV	21 (2.4)
Dopamine	IV	18 (2.1)
Milrinone	IV	18 (2.1)
Midazolam	IV, PO ^a^, buccal	17 (1.9)
Liposomal amphotericin B	IV	16 (1.8)
Insulin Aspart	IV, SC	14 (1.6)
Hypertonic dextrose (20% or greater)	IV, PO ^a^	14 (1.6)
Norepinephrine	IV	12 (1.4)
Epinephrine	IM, IV, local anaesthesia ^a^	11 (1.3)
Insulin (human, biosynthetic)	IV	11 (1.3)
Propofol	IV	10 (1.1)
Doxorubicin	IV	9 (1.0)
Potassium phosphates concentrate	IV	9 (1.0)
Dexmedetomidine	IV, IN ^a^	8 (0.9)
Mercaptopurine	PO	8 (0.9)
Pegaspargase	IM, IV	8 (0.9)
Tramadol	IV, PO	8 (0.9)
Amino-acid infusion	IV	7 (0.8)
Diazepam	IV, PO	7 (0.8)
Insulin Detemir	SC	6 (0.7)
Esketamine	IV, PO ^a^	6 (0.7)
Levosimendan	IV	6 (0.7)
Lorazepam	IV, PO	6 (0.7)
Others ^b^	various	64 (7.3)
**Total**		**872 (100.0)**

*IA* intra-arterial, *IH *inhalation, *IM* intramuscular, *IN* intranasal, *IT* intrathecal, *IV* intravenous, *PO* oral, *SC* subcutaneous, *SL* sublingual

^a^Off-label administration route. ^b^A heterogenous group of different medications (*n* = 37)

**Table 3:** All identified high-alert medications (*n* = 872) in the sub-sample of medication error reports (*n* = 743) were divided into 14 level 2 Anatomical Therapeutic Chemical (ATC) code groups and further into 26 level 3 ATC code groups

ATC group	Medications	n (%)
**Blood substitutes and perfusion solutions (B05)**		**345 (39.6)**
IV solutions (B05B)	PN, lipid emulsion, hypertonic dextrose (20% or greater), amino acids	172 (19.7)
IV solution additives (B05X)	Hypertonic sodium chloride (> 0.9%), potassium chloride^a^, potassium phosphates^a^, magnesium sulfate^a^	170 (19.5)
Peritoneal dialytics (B05D)	Bicavera®	2 (0.2)
Hemodialytics and hemofiltrates (B05Z)	Prismocal®	1 (0.1)
**Antineoplastic agents (L01)**		**139 (15.9)**
Antimetabolites (L01B)	Cytarabine, mercaptopurine, thioguanine, azacitidine, fludarabine, clofarabine, methotrexate (IV)	63 (7.2)
Plant alkaloids and other natural products (L01C)	Vincristine, vinblastine, etoposide	39 (4.5)
Cytotoxic antibiotics and related substances (L01D)	Doxorubicin, daunorubicin, dactinomycin, idarubicin	17 (1.9)
Other antineoplastic agents (L01X)	Pegaspargase, carboplatin, tretinoin, asparaginase Erwinia, cisplatin	14 (1.6)
Alkylating agents (L01A)	Treosulfan, busulfan, cyclophosphamide, temozolomide	5 (0.6)
Protein kinase inhibitors (L01E)	Selumetinib	1 (0.1)
**Analgesics (N02)**		**98 (11.2)**
Opioids (N02A)	Fentanyl (SL), morphine, oxycodone, tramadol	98 (11.2)
**Antithrombotic agents (B01)**		**75 (8.6)**
Antithrombotic agents (B01A)	Heparin, enoxaparin, warfarin, urokinase, antithrombin	75 (8.6)
**Cardiac therapy (C01)**		**68 (7.9)**
Cardiac stimulants excl. cardiac glycosides (C01C)	Dopamine, milrinone, norepinephrine, epinephrine, levosimendan	65 (7.5)
Antiarrhythmics, class I and III (C01B)	Amiodarone	3 (0.3)
Other cardiac preparations (C01E)	Adenosine	1 (0.1)
**Anesthetics (N01)**		**48 (5.5)**
Anaesthetics, general (N01A)	Propofol, esketamine, ketamine, thiopental, fentanyl (IV)	45 (5.2)
Anaesthetics, local (N01B)	Ropivacaine	3 (0.3)
**Psycholeptics (N05)**		**39 (4.5)**
Hypnotics and sedatives (N05C)	Midazolam, dexmedetomidine	25 (2.9)
Anxiolytics (N05B)	Diazepam, lorazepam, oxazepam	14 (1.6)
**Drugs used in diabetes (A10)**		**32 (3.7)**
Insulins and analogues (A10A)	Insulin aspart, insulin (human, biosynthetic), insulin detemir, insulin glulisine	32 (3.7)
**Other therapeutic groups (*n* = 6)**		**27 (3.1)**
**Total**		**872 (100.0)**

*IV* intravenous, *PN* parenteral nutrition, *SL* sublingual

^a^ Concentrated solution

The active substances and administration routes of each identified high-alert medication (*n* = 71 different drugs with a total on 872 high-alert medications) are presented in Table 3. The most frequently mentioned drugs were parenteral nutrition preparations (14.9%), hypertonic sodium chloride (10.7%), potassium chloride concentrate (7.6%), morphine (5.4%), and heparin (4.9%). These TOP 5 substances accounted for over 40% of all identified high-alert medications (*n* = 872).

### Administration routes of the identified high-alert medications

**Fig. 2:**
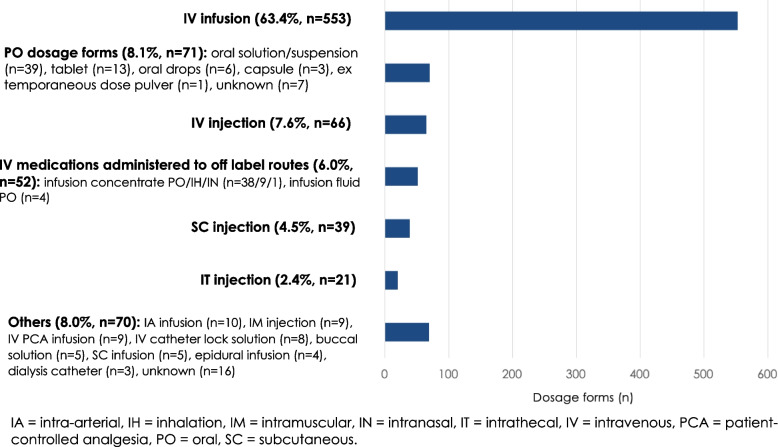
Administration routes and dosage forms of high-alert medications (*n* = 872) was identified in the sub-sample (*n* = 743 medication error reports)

Over 70% (*n* = 619/872, 71%) of the high-alert medications identified in the sub-sample (*n* = 743 ME reports) were administered intravenously (e.g., infusion, injection, patient-controlled analgesia (PCA), or catheter lock solution) (Fig. 2). The second most common route of administration was oral, where the range of drug formulations was diverse. In addition to the official route of administration, intravenous preparations were also administered by other routes, such as orally (e.g., midazolam as procedural pre-medication, concentrated electrolytes to correct electrolyte deficiencies in young children, and glucose 30% as pain relief in new-borns), inhaled (e.g., sodium chloride concentrate to produce sputum) and intranasally (e.g., dexmedetomidine for minimal sedation) (Fig. 2, Table 2). There was a shortage of a commercial local anaesthetic containing both lidocaine and epinephrine during the study period, which is why this solution needed to be prepared manually by combining the intravenous preparations of these drugs (Table 2).

### High-alert medications associated with the most serious ME reports

Of all ME reports included in the study sample (*n* = 2,132), 1.3% (*n* = 28) were rated to the two highest risk classifications (IV = significant risk and V = serious risk) and/or resulted in severe harm to the patient. Of these, 16 (57.1%) were associated with high-alert medications, with 12 ME reports rated into the highest risk classifications (IV and V), two ME reports that had caused severe harm to the patient and two ME reports meeting both inclusion criteria (Table 4). The TOP 3 ATC groups involved in the most serious MEs were analgesics (N02) (*n* = 8), antineoplastic agents (L01) (*n* = 3), and antithrombotic agents (B01) (*n* = 3). The most appearing individual medication was morphine (*n* = 6).

**Table 4:** The most serious medication errors involving high-alert medications (*n* = 18) within the sub-sample (*n* = 16/743 medication error reports)

High-alert medication	Short description of medication errors
Morphine (*n* = 6, 33.3%)	• Infusion rate programmed 12.5 mL/h instead of 2.5 mL/h • A full daily dose prescribed six times, although the daily dose should have been divided into six doses • PO dose accidentally given IV • Three IV doses given within 30 min prior to the transfer resulted in the deterioration of the patient’s condition in the receiving unit • The patient received accidentally another patient's medicine • Morphine IV infusion prescribed and given at 3.5 mL/h instead of 0.35 mL/h
Aspartinsulin (*n* = 2, 11.1%)	• CVC blood glucose samples were contaminated by glucose infusion, which led to unnecessary dose increases of IV infusion and hypoglycemia • The changes made into the insulin pump were not approved, which resulted in a new order on incorrect grounds the next day
Enoxaparin (*n* = 2, 11.1%)	• A fivefold dose, because the dose was prepared from the undiluted medicine (100 mg/ml) instead of the diluted one (20 mg/ml) • The dose was decreased to from 20 to 10 mg (no prefilled syringe available), but a 100 mg syringe was mistakenly prescribed
Oxycodone (*n* = 2, 11.1%)	• PO dose prescribed to IV route • A respiratory arrest resulting from a combination of too many PCA boluses and epidural analgesia
Carboplatin (*n* = 1, 5.6%)	• Too rapid etoposide infusion (1 h instead of 3 h) because of a mix-up between infusion times
Etoposide (*n* = 1, 5.6%)	
Dopamine (*n* = 1, 5.6%)	• 100-fold infusion rate because of pump programming error (23 mL/h instead of 0.23 mL/h)
Heparin flush (*n* = 1, 5.6%)	• Accidental administration of parenteral nutrition to IA line after a mix-up between infusion syringes
Parenteral nutrition (*n* = 1, 5.6%)	
Vincristine (*n* = 1, 5.6%)	• An extra dose given to a patient suffering from neuropathy, because the previous dose was recorded in the wrong place

*CVC* central venous catheter, *IA* intra-arterial, *IV* intravenous, *PCA* patient-controlled analgesia, *PO* oral

### Comparison of ME severity between high-alert medications and other medications

A Pearson Chi-Square (χ2) of independence was performed to evaluate the relationship between consequences to the patient and medications involved in the ME reports (Table 5). The relationship between these variables was significant, χ2 (5, *N* = 2,132) = 17,151, *p* = 0.004. ME reports involving high-alert medications (*n* = 743) were more likely to cause any degree of harm (minor, moderate and severe) to the patient when compared to ME reports involving other medications (*n* = 1,389) within the study sample (*n* = 2,132). The same statistical test was performed to evaluate the relationship between ME risk classification and medications involved in ME reports. The relationship between these variables was also significant, χ2 (5, *N* = 2,132) = 46,669, *p* = 0.000. ME reports involving high-alert medications (n = 743) were more likely classified to highest risk classifications (IV-V) than ME reports involving other medications (*n* = 1,389).

**Table 5:** Patient harm and risk classification associated with medication error (ME) reports involving high-alert medications (*n* = 743) and other medications (*n* = 1,389)

	**ME severity**	**ME reports involving other medications n (%)**	**ME reports involving high-alert medications n (%)**	**Total n (%)**
Consequences to the patient	No harm	707 (50.9)	347 (46.7)	1054 (49.4)
	Minor harm	208 (15.0)	135 (18.2)	343 (16.1)
	Moderate harm	24 (1.7)	27 (3.6)	51 (2.4)
	Severe harm	1 (0.1)	4 (0.5)	5 (0.2)
	Not known	206 (14.8)	110 (14.8)	316 (14.8)
	Not reported	243 (17.5)	120 (16.2)	363 (17.0)
	Total	1,389 (100.0)	743 (100.0)	2,132 (100.0)
	Chi-square test	*X*^2^ (**5**, *N* = **2,132**) = 17,151, *p* = .004		
Risk classification	I (insignificant risk)	111 (8.0)	39 (5.2)	150 (7.0)
	II (low risk)	851 (61.3)	427 (57.5)	1278 (59.9)
	III (moderate risk)	308 (22.2)	237 (31.9)	545 (25.6)
	IV (significant risk)	10 (0.7)	11 (1.5)	21 (1.0)
	V (serious risk)	1 (0.1)	4 (0.5)	5 (0.2)
	Not reported	108 (7.8)	25 (3.4)	133 (6.2)
	Total	1,389 (100.0)	743 (100.0)	2,132 (100.0)
	Chi-square test	*X*^2^ (**5**, *N* = **2,132**) = 46,669, *p* = .000		

## Discussion

To the best of our knowledge, this study is both the largest analysis of MEs related to high-alert medications in a paediatric hospital to date and one of the first studies to show that the use of ISMP high-alert medications is associated with a risk for patient harm in paediatric hospital settings. While previous studies investigating high-alert medications from children's perspective have focused on identifying paediatric high-alert medications [17–21], it is equally important to study MEs associated with these drugs to determine the appropriate medication safety measures. This point represents an important area of research covered only in two previous publications, one with more limited study material [14] and the other focusing on the NICU setting [12]. In our study, more than 70 individual ISMP high-alert drugs were present in MEs, demonstrating the complexity and extensive skill requirements of medication safety management in paediatric university hospital settings. In comparison, other studies aiming to identify paediatric high-alert medications have included approximately 5–44 individual drugs or medication groups in their investigation [17–21]. In our study, the most common high-alert medications comprised parenteral nutrition, concentrated electrolytes, antineoplastic agents, opioids, and antithrombotic agents, which have also been highlighted in other studies investigating paediatric MEs [12, 14, 30, 31].

As expected, we found that a larger proportion of MEs related to high-alert medications were associated with serious harm to the patient than reports related to other drugs. This result corresponds to the findings of earlier paediatric studies [12, 14] and ISMP’s definition of high-alert medications [9]. However, the number and proportion of MEs associated with serious harm in our study were limited compared to other publications, perhaps because of the low reporting activity of physicians who most typically report severe errors [7, 12, 31, 32]. We also found that ME reports on high-alert medications were more likely to receive a higher risk classification, considering the harm's severity and the probability of recurring similar cases. Therefore, establishing safeguards promoting the safe use of high-alert medications throughout the MMU process is essential to ensure medication safety in paediatric hospitals [3, 9, 15, 16, 33]. Especially powerful error-reduction strategies focusing on changes to the system where individuals operate are recommended, which is contrary to conventional easy-to-implement defences relying mostly on human vigilance (e.g., awareness, manual double checks, staff education, and appeals to “be careful”) [4, 16, 34]. However, many of the most effective safeguards (e.g., electronic health records and clinical decision support) are designed for adults and have limited effectiveness in reducing paediatric-specific errors, so implementation of new technology requires systematic risk management and paediatric customisation [33, 35, 36].

In our study, the most serious MEs related to high-alert medications were associated with opioids, antineoplastic agents, antithrombotic agents, and insulin. These drugs have also been observed to cause severe paediatric MEs in other studies [7, 12, 14, 31, 37]. Although we found common MEs related to parenteral nutrition and concentrated electrolytes, supporting the previous evidence [12, 30, 31, 38], the number of serious MEs remained low. However, several paediatric studies have identified these drugs as high-alert medications [17–21]. Parenteral nutrition is associated with serious adverse events, such as infections and even deaths resulting from product contamination, complications concerning intravenous access (e.g., thrombosis, bloodstream infection) and metabolic homeostasis (e.g., hyper- or hypoglycaemia, fluid, and electrolyte disorders) [39, 40]. Likewise, too-concentrated peripheral potassium infusions are associated with the risk of necrosis. At the same time, too-rapid infusion rates or accidental administration of undiluted potassium chloride concentrate can lead to severe arrhythmias and cardiac arrest [19, 41]. Moreover, medication safety risks related to other concentrated electrolytes, such as sodium chloride, magnesium, calcium, and phosphate preparations, have also been highlighted elsewhere [15, 19, 40]. Overall, intravenous fluids and parenteral nutrition are very complex in composition and prescribed individually to each paediatric patient, which creates several risks for errors within the MMU process [39, 40, 42].

Over 70% of all identified high-alert medications and over 60% of the most severe MEs related to high-alert medications involved intravenous administration route in our study. The high prevalence of intravenous drugs has also been highlighted in other studies investigating paediatric MEs [5, 14, 37]. Numerous intravenous drugs have been included in paediatric high-alert medication lists [18, 19, 21]. This emphasises the introduction of safeguards to secure intravenous drug administration, such as more advanced clinical decision support systems, standardised ready-to-use infusions, barcode medication administration, and smart infusion pumps, which would preferably be integrated into the electronic health record system [17, 21, 33, 43, 44]. We also found that intravenous high-alert medications were administered to off-label routes (e.g., orally, intranasally, or inhaled) due to the lack of commercial products designed and registered for these administration routes. In Europe, efforts have been made to facilitate the development and availability of paediatric medicines by implementing the Pediatric Regulation (EU 1901/2006) in 2007 [45]. Still, there need to be more medications across many therapeutic areas and age groups (e.g., age-appropriate formulations and adequate dosing), and a significant proportion of the new paediatric medications might not be marketed in all countries [46]. Overall, the prevalence of orally administered drugs within our study material remained low, probably because the ISMP's list of high-alert medications in acute care settings focuses on parenteral drugs [9].

There are some limitations to the study. First, our study material consisted of self-reported MEs, which have been associated with the risk of underreporting [32, 47]. Nurses' significantly higher reporting activity may have contributed to the prominence of administration errors in our study sample, while prescribing errors have been found as common in paediatric hospitals [32, 48, 49]. Moreover, healthcare professionals might consider reporting of MEs involving high-alert medications more crucial than MEs related to other drugs. There was also variation in reporting activity between different care units and specialities, with the highest number of ME reports from the neonatal intensive care and oncology. The COVID-19 pandemic has also potentially affected the reporting activity of MEs, paediatric patient profile and the utilization rate of different high-alert medications in 2020. Second, the STROBE checklist for cross-sectional studies has been developed primarily for clinical trials involving medical interventions and patients as study material, which is methodologically different from studies applying medication safety incident data [22]. Consequently, some items, especially recommendations regarding the methods and results, required modification to apply to our study (Supplementary File 1). However, we found the modified STROBE checklist useful to support conducting and reporting the present type of study design.

Our results can be applied as a basis for risk management activities in paediatric hospitals alongside other studies investigating paediatric high-alert medications [12, 14, 17–21]. The present findings might be generalisable, at least to some extent, to the entire MMU process in paediatric care settings, involving also other drugs than high-alert medications. However, qualitative studies carried out by healthcare professionals in collaboration with human factor and ergonomic specialists still need to investigate the underlying root causes and contributing factors to fully understand MEs related to high-alert drugs in paediatrics. It would also be useful to take a closer look at the near-misses involving high-alert medications so that the identified safety risks could be addressed before they cause harm to patients. In further studies, more evidence on the effectiveness of different safeguards in the paediatric MMU process related to high-alert drugs needs to be produced, as only a few studies have addressed this issue so far [17, 21].

## Conclusions

The present study provides an overview of MEs related to high-alert medications at a paediatric university hospital over three years. While being common in ME reports and more likely to be associated with severe patient harm and risk of re-occurrence, preventive risk management actions should be targeted on high-alert medications in paediatric settings. Especially safeguarding the MMU process of intravenous drugs, such as parenteral nutrition, concentrated electrolytes, analgesics, and antineoplastic agents, might need to be prioritised. Special emphasis should also be placed on off-label use of high-alert medications as a key risk factor. Further research is needed to investigate the underlying root causes of MEs related to high-alert medications and the effectiveness of different safeguards in preventing these errors.

### Supplementary Information

**Additional file 1.** STROBE Statement—Checklist of items that should be included in reports of cross-sectional studies with modifications applied to this study.

**Additional file 2.** Characteristics of the medication error (ME) reports involving high-alert medications included in the sub-sample (*n*=743) and ME reports involving other medications (*n*=1,389). 

## Data Availability

The HaiPro medication error report data that support the findings of this study are available from Helsinki University Hospital (HUS), but restrictions apply to the availability of these data, which were used under license for the current study, and so are not publicly available. Data are, however, available from the authors upon reasonable request and with permission of HUS.

## Abbreviations

ATC code: Anatomical Therapeutic Chemical classification system for medications
CVC: Central venous catheter
HaiPro: Patient safety incident reporting system used by the study hospital
HUS: HUS Helsinki University Hospital
ISMP: Institute for Safe Medication Practices
IA: Intra-arterial
IH: Inhalation
IM: Intramuscular
IN: Intranasal
IT: Intrathecal
IV: Intravenous
ME: Medication error
NCC MERP: National Coordinating Council for Medication Error Reporting and Prevention
NICU: Neonatal intensive care unit
PCA: Patient-controlled analgesia
PO: Oral
PN: Parenteral nutrition
SC: Subcutaneous
SL: Sublingual
STROBE: Strengthening the Reporting of Observational Studies in Epidemiology
