# Supplementary material for: Medication errors related to high-alert medications in a paediatric university hospital – a cross-sectional study analysing error reporting system data
Source: BMC Pediatr. 2023 Oct 31;23:548. doi: 10.1186/s12887-023-04333-2 (PMC10617051; doi:10.1186/s12887-023-04333-2)
Supplement: Supplementary file 1 — Additional file 1. STROBE Statement—Checklist of items that should be included in reports of cross-sectional studies with modifications applied to this study. [file 12887_2023_4333_MOESM1_ESM.docx]

**Supplementary File 1. STROBE Statement—Checklist of items that should be included in reports of cross-sectional studies with *modifications applied to this study*.**

|  | Item No | Recommendation | *Modifications applied to this study* | Page No (and Chapter and Figure/Table) |
| --- | --- | --- | --- | --- |
| **Title and abstract** | 1 | (*a*) Indicate the study’s design with a commonly used term in the title or the abstract | - | 1 (Title) |
|  |  | (*b*) Provide in the abstract an informative and balanced summary of what was done and what was found | - | 1 (Abstract) |
|  | | Introduction | | |
| Background/rationale | 2 | Explain the scientific background and rationale for the investigation being reported | - | 2 (Background) |
| Objectives | 3 | State specific objectives, including any prespecified hypotheses | - | 2 (Background) |
|  | | Methods | | |
| Study design | 4 | Present key elements of study design early in the paper | - | 2 (Study Design) |
| Setting | 5 | Describe the setting, locations, and relevant dates, including periods of recruitment, exposure, follow-up, and data collection | Describe the setting, locations, and relevant dates, including periods of data collection *(no patients involved, as the study material consisted of self-reported medication errors)* | 2–4 (Study Setting, Figure 1, Data Collection and Processing) |
| Participants | 6 | (*a*) Give the eligibility criteria, and the sources and methods of selection of participants | (*a*) Give the eligibility criteria, and the sources and methods of selection of study material *(medication error reports)* | 2–4 (Figure 1, Table 1, Data Collection and Processing) |
| Variables | 7 | Clearly define all outcomes, exposures, predictors, potential confounders, and effect modifiers. Give diagnostic criteria, if applicable. | Clearly define all outcomes and exposures *(description of the key definitions and characteristics of medication errors, the study-material consisted of register-based self-reported medication errors)* | 2–4 (Figure 1, Table 1, Data Collection and Processing) |
| Data sources/ measurement | 8* | For each variable of interest, give sources of data and details of methods of assessment (measurement). Describe comparability of assessment methods if there is more than one group. | For each variable of interest, give sources of data and details of methods of assessment (measurement) *(description of the medication error report classification system in HaiPro, classification process in Helsinki University Hospital, and all manual classification tasks conducted as part of data processing)* | 2 and 4 (Data Collection and Processing) |
| Bias | 9 | Describe any efforts to address potential sources of bias | Describe any efforts to address potential sources of bias | 2 and 4 (Data Collection and Processing) |
| Study size | 10 | Explain how the study size was arrived at | Explain how the study size was arrived at |  |
| Quantitative variables | 11 | Explain how quantitative variables were handled in the analyses. If applicable, describe which groupings were chosen and why | - | 4–5 (Descriptive Quantitative Analysis, Statistical Analysis) |
| Statistical methods | 12 | (*a*) Describe all statistical methods, including those used to control for confounding | (*a*) Describe all statistical methods *(no statistical methods used to control for confounding)* | 4–5 (Statistical Analysis) |
|  |  | (*b*) Describe any methods used to examine subgroups and interactions | - | 4–5 (Statistical Analysis) |
|  |  | (*c*) Explain how missing data were addressed | (c) Explain how missing data were addressed | 4–5 (Statistical Analysis) |
|  |  | (*d*) If applicable, describe analytical methods taking account of sampling strategy | N/A *(analytical methods taking account of sampling strategy were not used)* | - |
|  |  | (*e*) Describe any sensitivity analyses | N/A *(sensitive analysis was not performed)* | - |
|  | | Results | | |
| Participants | 13* | (a) Report numbers of individuals at each stage of study—eg numbers potentially eligible, examined for eligibility, confirmed eligible, included in the study, completing follow-up, and analysed | (a) Report numbers of *medication error reports* at each stage of study—eg numbers potentially eligible, examined for eligibility, confirmed eligible, included in the study, and analysed *(the study material consisted of medication error reports instead of people, follow-up was not applicable)* | 3 (Figure 1) |
|  |  | (b) Give reasons for non-participation at each stage | (b) Give reasons for exclusion at each stage | 3 (Figure 1) |
|  |  | (c) Consider use of a flow diagram | (c) Consider use of a flow diagram | 3 (Figure 1) |
| Descriptive data | 14* | (a) Give characteristics of study participants (eg demographic, clinical, social) and information on exposures and potential confounders | (a) Give characteristics of *study material (medication error reports, e.g., nature of the event, case notifier, location, type of the incident)* | 5 (Characteristics of the ME reports comprising high-alert medications), Supplementary File 2 |
|  |  | (b) Indicate number of participants with missing data for each variable of interest | (b) Indicate number of *medication error reports* with missing data for each variable of interest | 3 (Figure 1), 9 (Table 5), Supplementary File 2 |
| Outcome data | 15* | Report numbers of outcome events or summary measures | Report numbers of outcome events *(sub-sample of high-alert medications: prevalence of different drugs, Anatomical Therapeutic Chemical (ATC) groups, administration routes, and the most severe outcomes; study sample: comparison of error severity between high-alert medications and other medications)* | 5–9 (High-alert medications involved in ME reports, Administration routes of the identified high-alert medications, High-alert medications associated with the most serious ME reports, Comparison of ME severity between high-alert medications and other medications, Tables 2–5, Figure 2) |
| Main results | 16 | *(*a) Give unadjusted estimates and, if applicable, confounder-adjusted estimates and their precision (eg, 95% confidence interval). Make clear which confounders were adjusted for and why they were included | N/A *(these were not measured)* | - |
|  |  | (*b*) Report category boundaries when continuous variables were categorized | N/A *(the research material included only discrete variables)* | - |
|  |  | (*c*) If relevant, consider translating estimates of relative risk into absolute risk for a meaningful time period | N/A *(these were not measured)* | - |
| Other analyses | 17 | Report other analyses done—eg analyses of subgroups and interactions, and sensitivity analyses | Report other analyses done *(descriptive quantitative analysis, frequencies and percentages)* | 5–9 (High-alert medications involved in ME reports, Administration routes of the identified high-alert medications, High-alert medications associated with the most serious ME reports, Comparison of ME severity between high-alert medications and other medications, Tables 2–5, Figure 2) |
|  | | Discussion | | |
| Key results | 18 | Summarise key results with reference to study objectives | - | 7 and 9–10 (Discussion, Conclusions) |
| Limitations | 19 | Discuss limitations of the study, taking into account sources of potential bias or imprecision. Discuss both direction and magnitude of any potential bias | - | 7 and 9–10 (Discussion) |
| Interpretation | 20 | Give a cautious overall interpretation of results considering objectives, limitations, multiplicity of analyses, results from similar studies, and other relevant evidence | - | 7 and 9–10 (Discussion, Conclusions) |
| Generalisability | 21 | Discuss the generalisability (external validity) of the study results | - | 7 and 9–10 (Discussion, Conclusions) |
|  | | Other information | | |
| Funding | 22 | Give the source of funding and the role of the funders for the present study and, if applicable, for the original study on which the present article is based | N/A *(This research received no external funding)* | 11 (Funding) |

*Give information separately for exposed and unexposed groups.

**Note:** An Explanation and Elaboration article discusses each checklist item and gives methodological background and published examples of transparent reporting. The STROBE checklist is best used in conjunction with this article (freely available on the Web sites of PLoS Medicine at http://www.plosmedicine.org/, Annals of Internal Medicine at http://www.annals.org/, and Epidemiology at http://www.epidem.com/). Information on the STROBE Initiative is available at [www.strobe-statement.org](http://www.strobe-statement.org).
