# Supplementary material for: Medication errors related to high-alert medications in a paediatric university hospital – a cross-sectional study analysing error reporting system data
Source: BMC Pediatr. 2023 Oct 31;23:548. doi: 10.1186/s12887-023-04333-2 (PMC10617051; doi:10.1186/s12887-023-04333-2)
Supplement: Supplementary file 2 — Additional file 2. Characteristics of the medication error (ME) reports involving high-alert medications included in the sub-sample (n=743) and ME reports involving other medications (n=1,389). [file 12887_2023_4333_MOESM2_ESM.docx]

**Supplementary File 2.** Characteristics of the medication error (ME) reports involving high-alert medications included in the sub-sample (n=743) and ME reports involving other medications (n=1,389).

| **Characteristic** | **n (%) of ME reports involving high-alert medications** | **n (%) of ME reports involving other medications** |
| --- | --- | --- |
| **Nature of the event** |  |  |
| Reached the patient | 469 (63.1) | 881 (63.4) |
| Near miss | 170 (22.9) | 356 (25.6) |
| Other patient safety observation | 104 (14.0) | 152 (11.0) |
| Total | 743 (100.0) | 1,389 (100.0) |
| **Case notifier** |  |  |
| Registered nurses | 647 (87.1) | 1,153 (83.0) |
| Physicians | 25 (3.4) | 56 (4.0) |
| Practical nurses | 13 (1.7) | 76 (5.5) |
| Allied healthcare team members (e.g., pharmacists) | 12 (1.6) | 28 (2.0) |
| Others | 6 (0.8) | 7 (0.5) |
| Not known | 40 (5.4) | 69 (5.0) |
| Total | 743 (100.0) | 1,389 (100.0) |
| **Type of unit where the ME happened** |  |  |
| Paediatric wards | 423 (56.9) | 850 (61.2) |
| Neonatal intensive care unit | 142 (19.1) | 210 (15.1) |
| Paediatric intensive care and monitoring unit | 68 (9.2) | 51 (3.7) |
| Anaesthesia and surgery | 39 (5.2) | 48 (3.4) |
| Home hospital | 39 (5.2) | 61 (4.4) |
| Day hospital and outpatient clinics | 16 (2.2) | 42 (3.0) |
| Paediatric emergency departments | 14 (1.9) | 64 (4.6) |
| Paediatric psychiatry | 2 (0.3) | 51 (3.7) |
| Others | 0 (0) | 12 (0.9) |
| Total | 743 (100.0) | 1,389 (100.0) |
| **Type of incident ^1^** |  |  |
| Administration error | 300 (38.3) | 629 (42.7) |
| Prescribing error | 160 (20.4) | 267 (18.1) |
| Preparation error | 73 (9.3) | 53 (3.6) |
| Transcribing error | 53 (6.8) | 129 (8.8) |
| Dispensing error | 51 (6.5) | 176 (11.9) |
| Error in ordering medication from the pharmacy | 31 (4.0) | 5 (0.3) |
| Error in medication storage | 27 (3.4) | 51 (3.5) |
| Error in the distribution of medication from the pharmacy | 12 (1.5) | 16 (1.1) |
| Error in patient counselling | 7 (0.9) | 20 (1.4) |
| Not known | 69 (8.8) | 127 (8.6) |
| Total | 783 (100.0) | 1,473 (100.0) |

^1^ One ME report can include more than one error.
